# Development and Characterization of PEGylated Fatty Acid-*Block*-Poly(ε-caprolactone) Novel Block Copolymers and Their Self-Assembled Nanostructures for Ocular Delivery of Cyclosporine A

**DOI:** 10.3390/polym14091635

**Published:** 2022-04-19

**Authors:** Ziyad Binkhathlan, Abdullah H. Alomrani, Olsi Hoxha, Raisuddin Ali, Mohd Abul Kalam, Aws Alshamsan

**Affiliations:** 1 Department of Pharmaceutics, College of Pharmacy, King Saud University, P.O. Box 2457, Riyadh 11451, Saudi Arabia; aomrani@ksu.edu.sa (A.H.A.); 436108203@student.ksu.edu.sa (O.H.); ramohammad@ksu.edu.sa (R.A.); makalam@ksu.edu.sa (M.A.K.); aalshamsan@ksu.edu.sa (A.A.); 2 Nanobiotechnology Research Unit, College of Pharmacy, King Saud University, P.O. Box 2457, Riyadh 11451, Saudi Arabia

**Keywords:** cyclosporine A, ethoxylated fatty acid, block copolymer, polymeric micelles, ocular

## Abstract

Low aqueous solubility and membrane permeability of some drugs are considered major limitations for their use in clinical practice. Polymeric micelles are one of the potential nano-drug delivery systems that were found to ameliorate the low aqueous solubility of hydrophobic drugs. The main objective of this study was to develop and characterize a novel copolymer based on poly (ethylene glycol) stearate (Myrj™)-*block*-poly(ε-caprolactone) (Myrj-*b*-PCL) and evaluate its potential as a nanosystem for ocular delivery of cyclosporine A (CyA). Myrj-*b*-PCL copolymer with various PCL/Myrj ratios were synthesized *via* ring-opening bulk polymerization of ε-caprolactone using Myrj (Myrj S40 or Myrj S100), as initiators and stannous octoate as a catalyst. The synthesized copolymers were characterized using ^1^H NMR, GPC, FTIR, XRD, and DSC. The co-solvent evaporation method was used to prepare CyA-loaded Myrj-*b*-PCL micelles. The prepared micelles were characterized for their size, polydispersity, and CMC using the dynamic light scattering (DLS) technique. The results from the spectroscopic and thermal analyses confirmed the successful synthesis of the copolymers. Transmission electron microscopy (TEM) images of the prepared micelles showed spherical shapes with diameters in the nano range (<200 nm). Ex vivo corneal permeation study showed sustained release of CyA from the developed Myrj S100-*b*-PCL micelles. In vivo ocular irritation study (Draize test) showed that CyA-loaded Myrj S100-b-PCL_88_ was well tolerated in the rabbit eye. Our results point to a great potential of Myrj S100-*b*-PCL as an ocular drug delivery system.

## 1. Introduction

**Figure 1:**
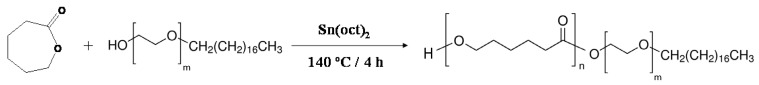
Synthetic scheme for Myrj-*b*-PCL copolymers.

Ethoxylated fatty acids e.g., PEG stearates (sold under the trademark Myrj™) are non-ionic surfactants widely used in various drug delivery systems (Figure 1). When intravenously injected, the PEG component of Myrj™ extends the circulation of the drug in plasma, while the fatty acid enhances the solubility of the fat-soluble drug. Depending on the length of PEG used, the ethoxylated fatty acid products have hydrophilic-lipophilic balance (HLB) values in the range of 11–18.8 [1].

The United States Food and Drug Administration (US FDA) has approved the use of Myrj™ products as safe excipients used in cosmetics, pharmaceutical formulations, and food additives [1]. Recently, there has been a growing interest in the use of Myrj™ products in the pharmaceutical industry. Several of these Myrj™ products have been used as absorption enhancers, emulsifiers, solubilizers, permeation enhancers, and stabilizers. Some derivatives of Myrj™ have also been utilized as inhibitors of P-gp to increase intestinal permeability and enhance the oral bioavailability of P-gp substrates [2,3,4].

Poly (ε-caprolactone) (PCL) is an eco-friendly polyester and has been widely utilized for tissue engineering and controlled drug delivery applications [5]. It allows homogenous distribution of drug molecules within the polymer matrix due to better compatibility with a variety of drugs [6]. Moreover, PCL exhibit, very slow degradation leading to prolonged drug release lasting for a few months [7]. The mechanical and physicochemical properties of PCL can easily be modified by blending or co-polymerization with different polymers. It has been found that co-polymerization of PCL with different polymers alters several intrinsic properties of PCL such as solubility, ionization, degradation pattern, and crystallinity resulting in a custom-made polymer with desired features for efficient drug delivery [8].

Polymeric micelles fabricated from PCL-based polymer were successful candidates for the delivery of lipophilic drugs such as valspodar, CyA, and paclitaxel [9,10,11]. Moreover, we recently showed that copolymerization of PCL with D–α–tocopheryl polyethylene glycol succinate (TPGS; with PEG MW > 2 kDa) was capable to self-assemble into nanocarriers with diameters smaller than 200 nm and critical association concentrations (CAC) in the nanomolar range [6]. More importantly, several derivatives of TPGS-*b*-PCL significantly improved the water solubility of paclitaxel (from ca. 0.3 µg/mL up to 88.4 µg/mL) and sustained the release of the loaded drug in vitro [6]. We demonstrated that paclitaxel-loaded TPGS_5000_-*b*-PCL_15000_ micellar formulation exhibited less than 10% drug release during the first 12 h, and approximately 36% cumulative drug release during 72 h in contrast to 61 and 100% paclitaxel release, respectively, from the marketed formulation (Ebetaxel^®^) [6].

Cyclosporine A (CyA) is a cyclic peptide and potent immunosuppressant agent prescribed for organ transplant patients (including heart, lung, and kidney) to prevent organ rejection. It is also very effective in the treatment of bone marrow transplants, systemic immune disorders, and various dermatological diseases (alopecia areata, psoriasis, pyoderma gangrenosum) [12]. Moreover, CyA is used to treat dry eye disease and uveitis [13]. However, CyA has poor corneal permeation owing to its rigid cyclic structure, high molecular weight, and barrier properties of the cornea [14]. For instance, after administration of a common eye-drop solution containing 0.05% CyA to treat dry eye syndrome, more than 95% of the drug reaches systemic circulation through trans-nasal or conjunctival absorption [15].

The main objective of the current study was to develop and characterize a novel copolymer based on poly (ethylene glycol) stearate (Myrj™)-*block*-poly(ε-caprolactone) (Myrj-*b*-PCL) and evaluate its potential as a nanosystem for ocular delivery of CyA.

## 2. Materials and Methods

### 2.1. Materials

Ethoxylated fatty acids [PEG-40 Stearate (commercially known as Myrj™ 52 or Myrj™ S40) and PEG-100 Stearate (commercially known as Myrj™ 59 or Myrj™ S100)], stannous octoate (95%), ε-Caprolactone, and HPLC-grade tetrahydrofuran (THF) were purchased from Sigma-Aldrich (St. Louis, MO, USA). Deuterated chloroform (CDCl_3_, 99.8%) was purchased from Cambridge Isotope Laboratories Inc. (Tewksbury, MA, USA). Acetonitrile (HPLC grade) was supplied by Fisher Scientific Co. (Leicestershire LE/15 RG, UK). Cyclosporine A (CyA) was purchased from Molekula Group LLC (Irvine, CA, USA). Potassium dihydrogen orthophosphate, dipotassium hydrogen orthophosphate, and potassium chloride were obtained from BDH Chemical Ltd. (Poole, England). All other chemicals were of analytical grade. Deionized water was prepared in-house using a Millipore system.

### 2.2. Methods

#### 2.2.1. Synthesis of Myrj-b-PCL Copolymers

Ring-opening polymerization of ε-caprolactone was the approach used to synthesize the copolymers [6,16]. For Myrj-*b*-PCL copolymer, either Myrj S40 (MW ~ 2000 Da) or Myrj S100 (MW ~ 4700 Da) were used as a macroinitiator and stannous octoate was used as the catalyst. Monomer (ε-caprolactone) to catalyst molar ratio was always kept at 1:500. Different ε-caprolactone to Myrj feed ratios were used to synthesize Myrj-*b*-PCL block copolymers with varying degrees of ε-caprolactone polymerization. Briefly, either Myrj S40 or Myrj S100, ε-caprolactone, and stannous octoate were added to an ampoule that was previously flamed and purged with nitrogen gas, which was then sealed under vacuum. The reaction was conducted at 140 °C for 4 h. Then, it was terminated by removing the reaction vessel (ampoule) from the oven and storing it at room temperature overnight.

#### 2.2.2. Characterization of the Myrj-b-PCL Copolymers

##### ^1^H Nuclear Magnetic Resonance (^1^H NMR) Spectroscopy

The products of the chemical reaction were evaluated by ^1^H NMR (Bruker Ultra shield 500.133 MHz spectrometer) in CDCL_3_.Tetramethylsilane (TMS) was used as an internal standard, and Topsin software was used to process the data and obtain the spectra. The number average molecular weight of all synthesized copolymers was determined from ^1^H NMR spectra by comparing the peak intensity of (–CH_2_CH_2_O–, δ = 3.65 ppm) present in the PEO segment of each copolymer to that of PCL (H–O–CH_2_–, δ = 4.07 ppm). The calculation used the integration area of the peaks of methylene protons of PCL at 4.07 ppm and of PEG at 3.65 ppm, respectively [6,16].

##### GPC Chromatography

Gel permeation chromatography system GPCmax (Malvern Panalytical Ltd., Malvern, OH, USA) and Viscotek Triple Detector Array 305 (Malvern, OH, USA) were used to determine the weight-average and number-average molecular weights as well as the molecular weight distribution of Myrj-*b*-PCL copolymers. A sample of 100 μL of the polymer from the prepared solution was injected into PLgel 5 μm MIXED-D, 7.5 × 300 mm (Agilent Technologies Inc., Santa Clara, CA, USA). The sample was pumped at a rate of 1 mL/min using THF as a mobile phase. The analysis was performed at 35 °C temperature. The data acquisition and processing were carried out by OmniSEC^TM^ (Version 4.7.0.406, Malvern Panalytical Ltd., Malvern, OH, USA) software. Prior to running the samples, GPC calibration was performed using EasiVial PS-M standards (Varian, Palo Alto, CA, USA).

##### Fourier-Transform Infrared (FTIR) Spectroscopy

The FTIR spectra of Myrj-*b*-PCL copolymers were recorded using an Alpha FTIR spectrophotometer (Bruker, Karlsruhe, Germany) equipped with Platinum Attenuated Total Reflectance (ATR) Module and diamond hemisphere. The instrument control as well as the data recording and processing are performed using OPUS version 7.8 (Bruker Optik GmbH, Ettlingen, Germany) software. Briefly, a small portion of the sample was kept on the sample holder covering the diamond hemisphere and the infrared (IR) beam was allowed to pass through the sample. The data were recorded in mid-infrared range starting from 4000 to 375 cm^−1^ with a spectral resolution of 2 cm^−1^. The software recorded the percentage transmittance with changing wave numbers to provide the infrared spectra.

##### Differential Scanning Calorimetry (DSC)

Thermograms of the Myrj-*b*-PCL copolymers were obtained utilizing DSC-60 (Shimadzu, Tokyo, Japan). Samples (3–5 mg) of the copolymers were loaded into aluminum pans. The samples were heated from 25–200 °C under nitrogen gas at a heating rate of 10 °C/min and purging at 40 mL/min along with scanning. Data analysis was conducted using the TA60 Version 2.10 (Shimadzu, Tokyo, Japan) thermal analysis software.

##### X-ray Diffraction (XRD)

The crystallinity state of the synthesized Myrj-*b*-PCL copolymers was studied using X-ray Diffractometer. Samples from the copolymers were loaded in the Ultima IV XRD instrument (Rigaku, Tokyo, Japan). The X-ray diffraction data of the samples were collected over the 3.0–50.0 degrees 2θ range at a scan speed of 0.50 degrees/min. The scanning process was done at room temperature.

##### Preparation of Drug-Free and CyA-Loaded Myrj-b-PCL Micelles

Micelles of Myrj-*b*-PCL block copolymers were prepared by the co-solvent evaporation method. Certain quantity of block copolymers (30 mg) was dissolved in 0.5 mL acetone. This preparation was added to a 3 mL purified water dropwise under stirring. The preparation was left on stirring overnight to evaporate the organic solvent. The particle size and polydispersity index of the self-assembled micelles were evaluated by dynamic light scattering (DLS) using Malvern Zetasizer™ 3000 (Malvern Instruments Ltd., Malvern, UK).

#### 2.2.3. Characterization of Myrj-b-PCL Micelles

##### Size, Polydispersity, and ζ-Potential

The particle size, polydispersity index, and ζ-potential of the self-assembled micelles were evaluated by dynamic light scattering (DLS) using Malvern Zetasizer™ 3000 (Malvern Instruments Ltd., Malvern, UK).

##### Critical Micelle Concentration (CMC)

The critical micelle concentration (CMC) of each block copolymer was evaluated by DLS as previously described [17,18]. The newly invented block copolymers were used to prepare micelles starting with the concentration of 500 µg/mL. For each block copolymer, subsequent two-fold serial dilutions by distilled water were applied to get various successive concentrations of a block copolymer. The intensity of the scattered light for the diluted samples of the prepared micelles was evaluated until reaching the levels where the intensity of the light scattering of the copolymer solution is similar to that of water. Zetasizer Nano ZS analyzer (Malvern Instruments Ltd., Malvern, UK) was used to measure the light scattering intensity as kilo counts per second (kcps) of each concentration of the block copolymers. The principle of this test is based on the fact that the readings of kcps remain constant at concentrations below CMC and start to abruptly increase at concentrations equal to CMC. All measurements were performed at 25 °C.

##### Drug Encapsulation Efficiency % and Drug Loading %

Samples (100 µL) of each prepared drug-loaded polymeric micelles after centrifugation at 13,000 rpm were diluted 100 times in acetonitrile and vortexed to disassemble the micelles and free the drug. The concentration of CyA in the supernatant was quantified using a previously published HPLC-UV assay [19]. Briefly, the HPLC system (Waters^TM^ 1500 series controller, Boston, MA, USA) is equipped with a wavelength detector (Waters^TM^ 2489a Dual^TM^ Absorbance detector, Boston, MA, USA), pump (Waters^TM^ 1525a Binary pump, Boston, MA, USA), and an automated sampling system (Waters^TM^ 2707 Plus Autosampler, Boston, MA, USA) monitored by “Breeze (Waters^TM^)” software. Sample (90 µL) containing CyA was analyzed using a mobile phase made of acetonitrile and purified water at a ratio of 75:25 running over C_18_ stationary phase (Macherey-Nagel, 4.6–150 mm, 10 µm particle size) maintained at 60 °C and 1.0 mL/min flow rate. The UV detector was set at 230 nm.

The encapsulation efficiency (%*EE*) and drug loading (%*DL*) of CyA in the prepared micelles were calculated using the following equations: Equations (1) and (2).

(1) $$EE\left(\%\right)=\frac{Amount\;of\;drug\;loaded\;\left(\mathrm{mg}\right)}{Amount\;of\;drug\;added\;\left(\mathrm{mg}\right)}\times 100,$$

(2) $$DL\left(\%\right)=\frac{Amount\;of\;drug\;loaded\;\left(\mathrm{mg}\right)}{Amount\;of\;polymer\;\left(\mathrm{mg}\right)+drug\;added\;\left(\mathrm{mg}\right)}\times 100,$$

##### Morphology

Morphology of the self-assembled structures was characterized by transmission electron microscopy (TEM). An aqueous droplet of micellar solution (20 uL) was placed on a copper-coated grid (Ted Pella, Inc., Redding, CA, USA). The grid was kept horizontally for 20 s to allow the colloidal aggregates to settle. A drop of 2% solution of phosphotungstic acid (PTA) in PBS (pH = 7.0) was then added to give the negative stain. After 1 min, the excess fluid was removed by using a strip of filter paper. The samples were left to get dry at room temperature and loaded into a JEM-1010 Transmission electron microscope (JEOL, Tokyo, Japan) operating at an acceleration voltage of 80 kV. Images were recorded with a high-speed read-out side-mounted MegaView^G2^ (Olympus, Hamburg, Germany) camera and processed with iTEM (Olympus Soft Imaging Solutions GmbH, Münster, Germany) software.

##### Ex Vivo Corneal Permeation

New Zealand rabbits weighing 2–3 kg were used in this study, and the protocol was approved by the Research Ethics Committee (REC) at King Saud University (No. SE-19-133). The cornea of rabbit eyes was excised and fixed between the donor and receptor components of the fabricated double jacketed transdermal diffusion cells (sampling system-SFDC 6, Logan, NJ, USA). The fixing of the cornea was done in such a way that the epithelial surface faced the donor compartment. Simulated-tear fluid (pH 7.4) with 0.5% (*w*/*v*) Tween-80 was filled in the receptor component of the diffusion cells. The water (37 °C) was allowed to flow in the outer jacket of the diffusion cells. The diffusion cells were placed on different stations of the LOGAN instrument. Continuous magnetic stirring could remove any air bubbles in the receptor component during sampling. Of each group (in triplicate), 500 μL CyA-containing formulations (CyA-micelles and Restasis^®^, 0.05%, *w*/*v*) were placed in the donor compartments. The samples from the receptor component were taken at predetermined time intervals for 4 h.

The CyA content was analyzed by the previously reported LC-MS/MS method with minor modifications [20,21,22]. Briefly, chromatographic separation of CyA was achieved with the help of Waters Acquity UPLC H-Class (Waters, Milford, MA, USA) equipped with the quaternary solvent manager, degasser, and column heater, sample manager having standard flow-through-needle and maximum injection volume of 10 µL. A Waters Acquity UPLC BEH™ C18 column (1.7 μm, 2.1 mm × 50 mm, Waters, Milford, MA, USA), maintained at 45 °C was utilized for drug elution. The mobile phase was eluted in isocratic mode at a flow rate of 0.3 mL/min, consisting of a mixture of 20 mm ammonium acetate and acetonitrile (20:80). Total run time was fixed to 3.5 min.

Mass spectrometric detection was performed using Waters Acquity TQD detector (Waters, Milford, MA, USA), equipped with an electrospray ionization (ESI) source, and operated in positive ionization mode. Selected ion recording (SIR) was used for the identification and quantification of CyA. For CyA, the SIR was performed at *m*/*z* 1202.85. The source and desolvation temperatures were set at 150 °C and 350 °C, respectively. Nitrogen was used as desolvation and nebulizing gas at a 600 L/h flow rate. The capillary and cone voltages were set at 3 kV and 95 V, respectively. The MassLynx software (Version 4.1) was used to control the UPLC–MS/MS system as well for data acquisition and processing.

Samples were centrifuged at 15,000 rpm for 10 min and filtered (0.45 µm) to remove any particulate matter. Precisely 100 µL of the filtered sample was mixed with 900 µL of mobile phase composition and vortexed for 30 s. The diluted samples were transferred to UPLC vials and 5 µL of the samples were injected into the UPLC-MS/MS system.

The permeation parameters including the flux (*J*) and the apparent permeability (Papp) were calculated from the slopes obtained by plotting the permeated amount of CyA (µg.cm^−2^) against time (*h*), using the following equations: Equations (3) and (4).

(3) dQdt=J (μgcm−2·h−1),

(4) JC0=Papp (cmh−1),

where (*Q*) signifies the amount of drug crossing the cornea, (*dQ*/*dt*) is the linear portion of the slope, (*t*) is the contact time of the formulation with the corneal surface, and (*C*_0_) is the initial concentration of the drug.

#### 2.2.4. In Vivo Ocular Irritation Test

All the procedures in this experiment were performed according to the guidelines of the Association for Research in Vision and Ophthalmology (ARVO) for animal use in ophthalmic and vision research. In accordance with the ARVO guidelines, only one eye (left eye) of all rabbits were chosen for testing purpose (the right eye was instilled with 0.9% NaCl as a negative control), and Draize’s eye test was used for assessing the ocular safety of CyA-loaded Myrj-*b*-PCL micelles [23]. Generally, for one test formulation, a maximum of six animals (rabbits) are required; however, this number can be decreased to three if there might be a chance of any severe ocular damage [24]. Thus, in this study, nine rabbits were divided into three groups each containing three (*n* = 3) for the irritation testing of the three formulations. Forty microliters (40 μL) of each formulation including blank were instilled into the lower conjunctival sac of each rabbit of the respective groups. All the rabbits in the conscious state received 3 consecutive instillations in the conjunctival sac of the left eye at an interval of 10 min for a short-term eye irritation test. After 1 h of the last dosing, eyes were periodically observed for any injuries or signs and symptoms in the cornea, iris and conjunctiva, photographs were captured, and scoring was done.

#### 2.2.5. Data Analysis

The collected data were reported as mean ± standard deviation (SD). Based on the number of groups being compared, statistical significance was analyzed either by Student’s *t*-test or one-way analysis of variance (ANOVA) followed by a post hoc test (LSD). The level of significance was set at α = 0.05.

## 3. Results and Discussion

### 3.1. Synthesis and Characterization of Myrj-b-PCL Copolymers

Table 1 shows the different Myrj-*b*-PCL copolymers synthesized. The copolymers were characterized by several analytical techniques including ^1^H NMR, GPC, FTIR, DSC, and XRD.

#### 3.1.1. ^1^H NMR

**Figure 2:**
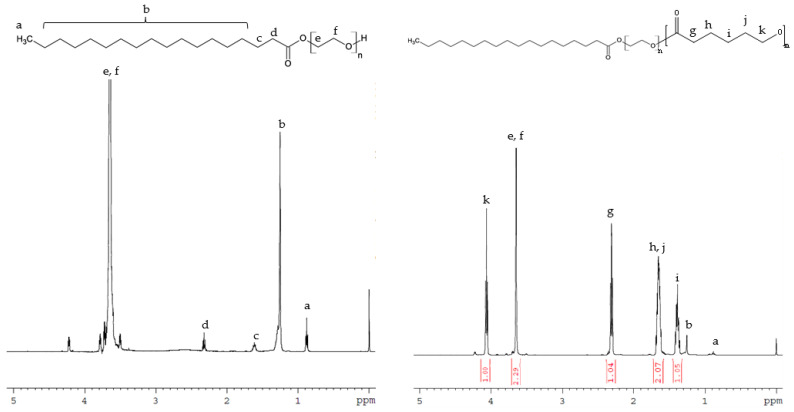
Representative ^1^H NMR spectra of Myrj S100 (**left**) and Myrj S100-*b*-PCL_88_ (**right**).

Representative ^1^H NMR spectra of Myrj S40 and Myrj S100 and the synthesized copolymers are shown in Figure 2 and Figure S1. Myrj-*b*-PCL copolymers show the following signals: δ = 4.07, 2.32, 1.67, and 1.38 ppm, which were assigned to (H–O–CH_2_–), (–CO–CH_2_–), (–CO–CH_2_–CH_2_–CH_2_–CH_2_–CH_2_–OH), and (–CO–CH_2_–CH_2_–CH_2_–CH_2_–CH_2_–OH) of the PCL segment, respectively. The peak at δ = 3.65 ppm was assigned to the methylene protons of the PEO in Myrj segment (CH_3_–O–CH_2_–CH_2_–O–). The lower peaks in the aliphatic region (δ = 0.87–1.28 ppm) belong to various moieties of stearate tails. The molecular weight and the composition of the synthesized copolymers were determined by ^1^H NMR based on the intensity ratio between the peaks at δ = 4.07 ppm and 3.65 ppm. ^1^H NMR data were found to be consistent with the theoretical values, which confirms the successful synthesis of Myrj-*b*-PCL copolymers.

#### 3.1.2. GPC

**Figure 3:**
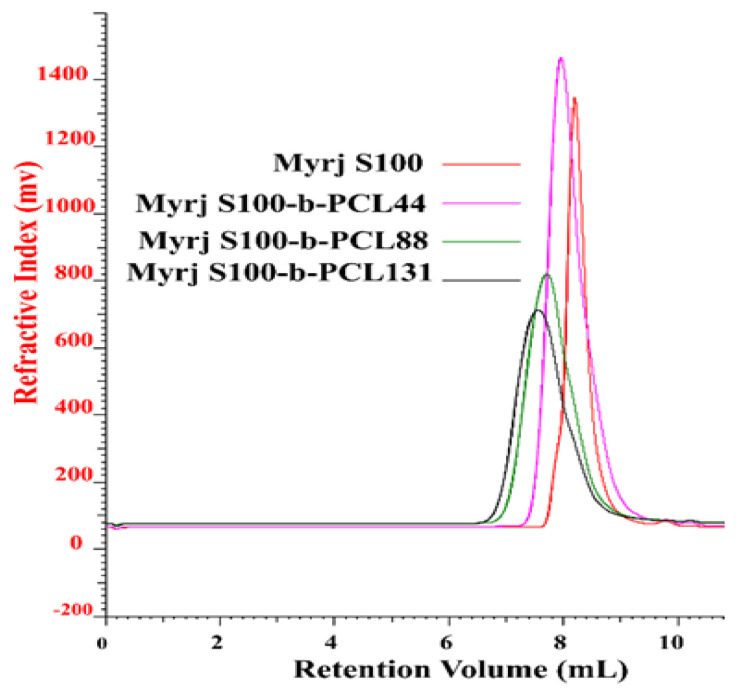
GPC chromatograms of unmodified Myrj S100 and the synthesized Myrj S100-*b*-PCL copolymers.

GPC analysis further confirmed the successful synthesis of Myrj-*b*-PCL with dispersity (Ð) ranging from 1.34–1.67. Figure 3 and Figure S2 show the GPC chromatograms of Myrj S40-*b*-PCL and Myrj S100-*b*-PCL copolymers, respectively. The retention volumes for Myrj S100 appeared at 8.0 mL. However, as expected, the peaks for all synthesized Myrj S100-*b*-PCL copolymers shifted to lower retention volumes (higher molecular weights). Each copolymer appeared as a single peak, which confirms that the polymerization reaction was successful. The molecular weights of the synthesized copolymers were calculated based on the prepared calibration curve using polystyrene standards. The GPC data for all the synthesized copolymers are presented in Table 1.

#### 3.1.3. FTIR

**Figure 4:**
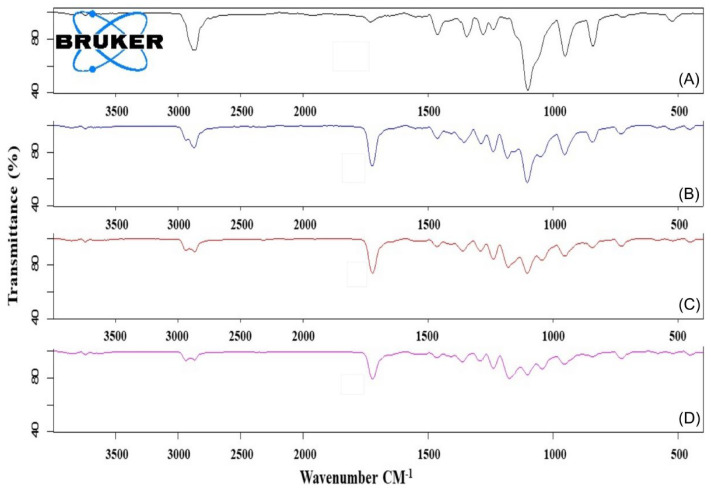
FTIR spectra of Myrj S100 (**A**), Myrj S100-*b*-PCL_44_ (**B**), Myrj S100-*b*-PCL_88_ (**C**), and Myrj S100-*b*-PCL_131_ (**D**).

FTIR spectra of Myrj S40 and Myrj S100 as well as their corresponding copolymers were shown in Figure 4 and Figure S3, respectively. For Myrj moieties, the characteristic bands for carbonyl groups (of stearate) appeared at 1737–1738 cm^−1^. For the synthesized Myrj-*b*-PCL copolymers, the carbonyl band was shifted to 1724–1734 cm^−1^ with stronger intensity due to the formation of PCL. Moreover, it was noticed that with increasing the molecular weight of PCL, the aliphatic CH stretching band of ε-CL at 2944–2945 cm^−1^ increased. On the other hand, the absorption band of CH stretching vibration in PEO and stearate moieties of Myrj at 2884–2885 cm^−1^ decreased. These observations verify the presence of intermolecular interactions and point to the formation of Myrj-*b*-PCL diblock copolymers. Indeed, a similar trend was previously reported for PEO-*b*-PCL copolymers [25].

#### 3.1.4. XRD

**Figure 5:**
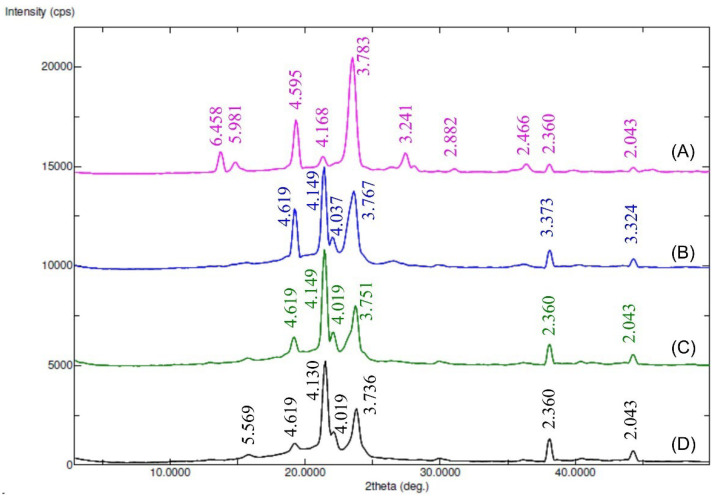
XRD diffractograms (peaks with d-values) of Myrj S100 (**A**), Myrj S100-*b*-PCL_44_ (**B**), Myrj S100-*b*-PCL_88_ (**C**), and Myrj S100-*b*-PCL_131_ (**D**).

The X-ray diffractograms of Myrj and the synthesized Myrj-*b*-PCL copolymers are shown in Figure 5 and Figure S4. The diffraction pattern of unmodified Myrj S40 and Myrj S100 exhibited good crystallinity with two sharp peaks appearing at 2θ = 19.3° and 23.4°, which are the characteristic crystalline peaks of PEO [26]. The synthesized copolymers showed different crystallinity behavior according to their PCL content. In addition to PEO peaks (of Myrj), the Myrj-*b*-PCL copolymers showed strong peaks at 2θ = 21.5° and 23.8° corresponding to the PCL crystalline units [25]. Furthermore, in all the synthesized Myrj-*b*-PCL copolymers, the sharp crystalline peak of PEO at 2θ = 19.3° was reduced while the intensity of the characteristic peaks of PCL increased with the increase in PCL molecular weight. These may be attributed to a conformational change, which affected the crystallinity of the PEG segment of Myrj upon increasing the PCL molecular weight.

#### 3.1.5. DSC

**Figure 6:**
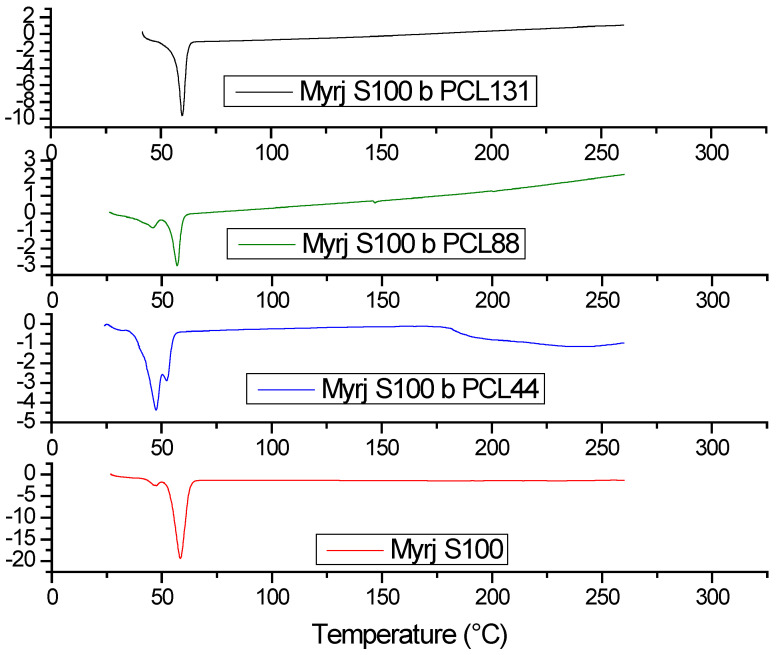
DSC thermograms of Myrj S100, Myrj S100-*b*-PCL_44_, Myrj S100-*b*-PCL_88_, and Myrj S100-*b*-PCL_131_.

Thermal properties of Myrj-*b*-PCL copolymers and their Myrj precursors were investigated by DSC. As shown in Figure 6 and Figure S5, DSC thermograms of Myrj S40, Myrj S40-*b*-PCL_18_, and Myrj S40-*b*-PCL_35_ exhibited strong peaks at 51.5 °C, 39.0 °C, and 50.5 °C, respectively, corresponding to their melting points. Moreover, DSC thermograms of Myrj S100, Myrj S100-*b*-PCL_44_, Myrj S100-*b*-PCL_88_, and Myrj S100-*b*-PCL_131_ exhibited strong peaks at 59.0 °C, 48.5 °C, 58.0 °C, 59.0 °C, respectively, corresponding to their melting points.

It was noticed that the melting temperature of Myrj increases with the increase in molecular weight of PEO (i.e., Myrj S40 *vs.* Myrj S100). Moreover, the melting temperatures of the Myrj-*b*-PCL copolymers correlate well with the PCL content. Specifically, the melting temperatures of the copolymers increased with the increase in the PCL molecular weight. Moreover, it was noticed that unmodified Myrj S40 and Myrj S100 each had a single sharp peak demonstrating a crystal pattern typical for the PEO crystal phase reported with PEO homopolymer [26]. Myrj-*b*-PCL copolymers, on the other hand, showed either broader and/or bimodal peaks (Figure 6). The broad or bimodal peaks are likely due to overlapped or separate endothermic peaks of Myrj (PEO) and PCL blocks. This is in agreement with the XRD results, where Myrj-*b*-PCL copolymers demonstrated a summation of both the PEO and PCL diffraction peaks. This indicates that both blocks could crystallize and form separate crystals, which were also previously observed with PEO-*b*-PCL copolymers [26].

### 3.2. Preparation and Characterization of Drug-Free and CyA-Loaded Myrj-b-PCL Micelles

**Figure 7:**
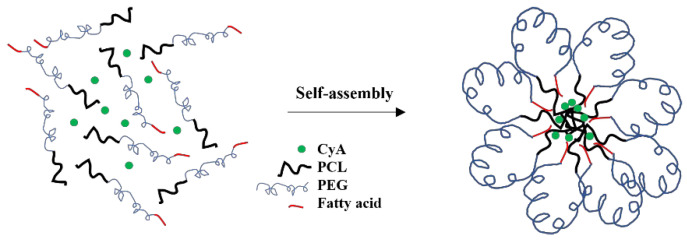
Proposed model for Myrj-*b*-PCL micelle formation in water.

Several water-miscible organic solvents were used (including acetonitrile, tetrahydrofuran, and acetone) to prepare micelles using the cosolvent evaporation method. Despite several attempts to prepare micelles using different organic solvent-to-water ratios as well as other methods of preparation (e.g., film-hydration method), none of the drug-free or CyA-loaded Myrj S40-*b*-PCL copolymers formed micelles with an acceptable yield or size. On the other hand, both Myrj S100-*b*-PCL_88_ and Myrj S100-*b*-PCL_131_ copolymers formed micelles with mean diameters <200 nm and with good yield (minimal or no precipitation). The only difference between Myrj S40 and Myrj S100 is the molecular weight PEG. Myrj S40 has 40 repeating units of PEG (i.e., PEG molecular weight = 1760 Da), whereas Myrj S100 comprised 100 repeating units of PEG (i.e., PEG molecular weight = 4400 Da). It seems that a PEG chain longer than 40 (MW > 1760 Da) was needed to provide the necessary folding to bring the stearate moiety (hydrophobic) in proximity to PCL, the core-forming block, during micelle formation in water. It is believed that this would be the thermodynamically favorable orientation for Myrj-*b*-PCL copolymer during the micellization process in the aqueous phase (Figure 7), which would be similar to the one reported for flower-like micelles formed from A-B-A triblock copolymers, where B is the hydrophilic block (e.g., Pluronic-R) [27]. In fact, this is similar to what we have observed and recently reported with α-tocopheryl polyethylene glycol succinate-*b*-PCL copolymers, where the micelles only formed when the molecular weight of PEG was higher than 2000 Da [6]. Nonetheless, this is only a hypothetical model that needs to be verified. Further studies are needed to investigate the conformation of these micelles in water (e.g., by using ^1^H NMR in D_2_O) [28,29] and to examine the morphology of the self-assembled structures in water (e.g., by using cryogenic TEM or atomic force microscopy) [30,31].

#### 3.2.1. Size, Polydispersity Index, and ζ-Potential

The particle size, polydispersity index (PDI), and ζ-potential of unloaded Myrj S100-*b*-PCL micelles are presented in Table 1. The mean particle size of the unloaded Myrj S100-*b*-PCL_44_, Myrj S100-*b*-PCL_88_, and Myrj S100-*b*-PCL_131_ micelles was 49.8, 70.3, and 72.6 nm, respectively. The PDI values of Myrj S100-*b*-PCL_88_ and Myrj S100-*b*-PCL_131_ were both found to be ≤0.21, which reflects good homogeneity of particle size distribution. Myrj S100-*b*-PCL_44_ micelles, however, showed a significantly higher PDI value (0.38 ± 0.14) compared to the other two. This is likely due to the presence of a bimodal population (Figure S6). It is also worth mentioning that there was significant polymer precipitation during the preparation of the micelles and after centrifugation. In the field of nano-drug delivery, a PDI ≤ 0.3 is considered optimal and indicates a homogenous population of the carrier system [32]. The ζ-potential values of all the Myrj S100-*b*-PCL micelles were nearly neutral to slightly negative, which is typical of polymeric micelles.

The particle size, PDI, and ζ-potential CyA-loaded Myrj S100-*b*-PCL are presented in Table 2. The size of the prepared micelles did not change significantly after drug loading except for Myrj S100-*b*-PCL_88_, where the CyA-loaded micelle size significantly increased (*p* < 0.05, paired Student’s *t*-test) compared to the unloaded counterpart. While CyA-loaded Myrj S100-*b*-PCL_88_ showed a uniform size with a relatively low PDI value (Table 2, Figure S7), Myrj S100-*b*-PCL_44_ and Myrj S100-*b*-PCL_131_ showed high variability in size and a relatively higher PDI value (Table 2). 

#### 3.2.2. CMC

The calculated CMC values for Myrj S100-*b*-PCL_88_ and Myrj S100-*b*-PCL_131_ micelles were 2.08 ± 0.08 and 2.70 ± 0.59 µM, respectively. The difference between the two CMC values was not statistically significant (Student’s *t*-test, *p* > 0.05). These values are much lower than the values reported for polymeric surfactants, which are usually in the millimolar range.

#### 3.2.3. Drug Encapsulation Efficiency (*EE*%) and Drug Loading (*DL*%) 

The drug loading% (*DL*%) and encapsulation efficiency% (*EE*%) of CyA-loaded micelles are presented in Table 2. Myrj S100-*b*-PCL_88_ showed a significantly higher *EE*% and *DL*% of CyA compared to Myrj S100-*b*-PCL_131_ (*p* < 0.05, Student’s *t*-test). Specifically, Myrj S100-*b*-PCL_88_ had an *EE*% of over 54% and *DL*% of 5.62%, which translates to an aqueous solubility of 540 µg/mL (i.e., a 2348-fold increase in CyA solubility). Although this loading level is lower than that previously reported for CyA in methoxy poly(ethylene oxide)-*block*-poly(ε-caprolactone) (PEO-*b*-PCL) micelles [19,21], it is sufficient to prepare the required concentration for ocular administration (0.05% *w*/*v*) [33]. Based on these results, only Myrj S100-*b*-PCL_88_ was selected for the next studies.

#### 3.2.4. Morphology

**Figure 8:**
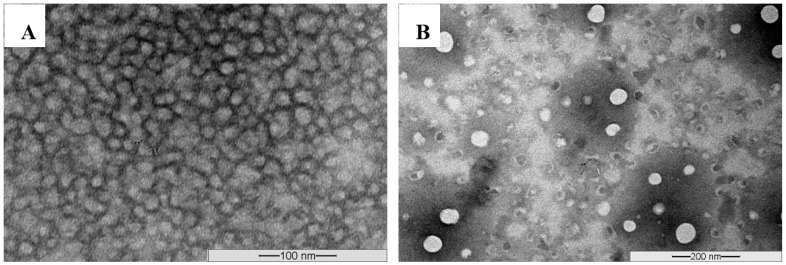
Representative TEM images obtained from unloaded Myrj S100-*b*-PCL_88_ (**A**) and CyA-loaded Myrj S100-*b*-PCL_88_ micelles (**B**) using JEOL JEM-1100 Transmission electron microscope (JAPAN) operating at an acceleration voltage of 80 kV.

The morphology of the unloaded, as well as CyA-loaded Myrj S100-b-PCL micelles, was studied by using transmission electron microscopy (TEM). The images revealed that these micelles were of spherical shape as shown in Figure 8.

### 3.3. Transcorneal Permeation of CyA-Loaded Myrj-b-PCL Micelles

**Figure 9:**
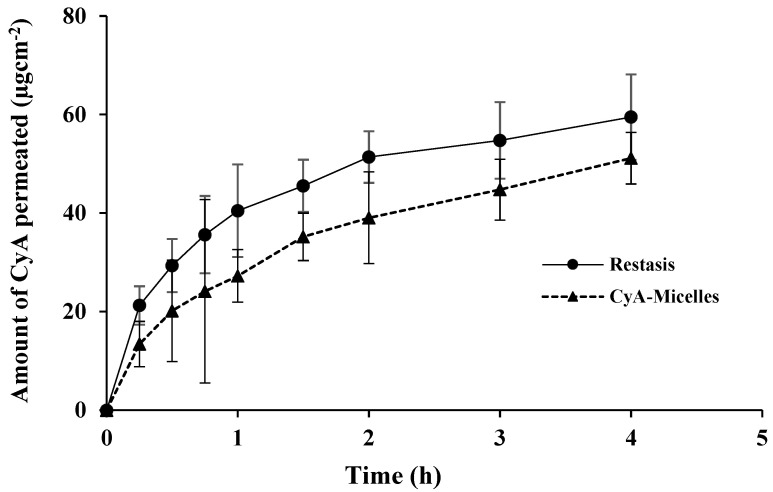
Transcorneal permeation of CyA from Myrj S100-*b*-PCL_88_ micelles and Restasis^®^. (Mean ± SD, *n* = 3).

The steady-state flux (*J*) and apparent permeability (*P_app_*) of CyA was calculated by considering the involved corneal area (0.5024 cm^2^), the volume of permeation medium (5.2 mL), and initial drug concentrations (500 µg/mL). In STF (pH 7.4), Tween-80 (0.5%, *w*/*v*) was added to improve the solubility of the highly lipophilic drug (CyA). From the plot shown in Figure 9, and the calculated values as summarized in Table 3, Myrj S100-*b*-PCL_88_ micelles exhibited a smooth and linear permeation of CyA as compared to Restasis^®^. The cumulative amounts of CyA permeated were 51.14 ± 5.23 and 59.49 ± 8.67 µg.cm^−2^ (at 4 h) from CyA-Micelles and Restasis^®^, respectively. The difference was not statistically significant (*p* > 0.05, Student’s *t*-test).

The permeation of CyA from Restasis^®^ seemed higher initially (up to 1 h) as compared to CyA-Micelles, but overall, the total permeated amount of CyA through the excised rabbit cornea was comparable for the two formulations (*p* > 0.05, Student’s *t*-test). From the permeation profile, it was concluded that CyA-Micelles may provide sustained delivery of the loaded CyA from the micelles compared to that of Restasis^®^.

### 3.4. In Vivo Ocular Irritation Test

The irritation potential (if any) of the blank and CyA-loaded Myrj S100-*b*-PCL_88_ micelles towards the anterior ocular segment was investigated for 24 h by following Draize’s test [23]. The observations were noted after the instillation of 40 µL (three times at an interval of 10 min) into the left eyes of all the New Zealand rabbits (*n* = 3) in comparison to the marketed CyA ocular formulation (Restasis^®^). Any changes were observed by visual examination of the cornea, iris, and conjunctiva of the treated eyes of all rabbits [34]. On the basis of signs and symptoms of ocular irritation (including redness, swelling, chemosis, edema, cloudiness, edema, hemorrhage, and or any discharge other than normal) which may arise in the treated eyes, the scoring was done as per the theoretical scores for grading system (Table S1) [35]. The irritation potential was categorized according to a classification system (Table S2) [36]. The obtained scores and signs of discomfort (if any) during the experiment for all tested formulations were recorded in Table 4.

**Figure 10:**
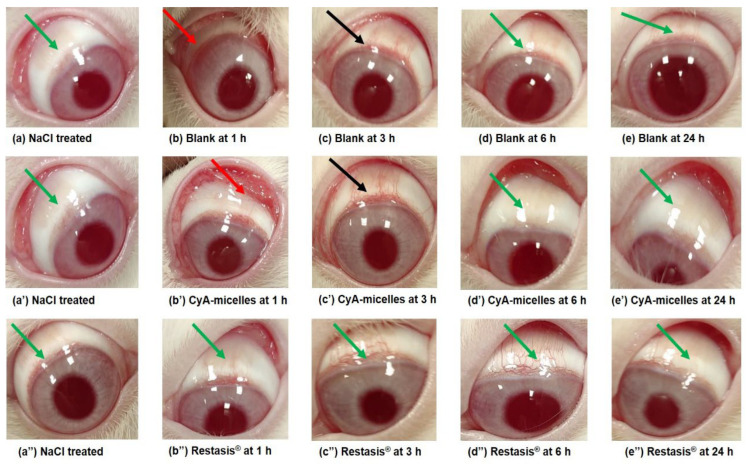
Photographs captured during irritation study in rabbit eyes. NaCl treated eyes of respective groups (**a**,**a′**,**a″**) (green arrows). Post instillation of blank Myrj S100-*b*-PCL_88_ micelles at 1 h (**b**) (red arrow); at 3 h (**c**) (black arrow); at 6 h (**d**) and at 24 h (**e**). Post instillation of CyA-loaded Myrj S100-*b*-PCL_88_ micelles (CyA-Micelles) at 1 h (**b′**) (red arrow); at 3 h (**c′**) (black arrow); at 6 h (**d′**) and at 24 h (**e′**). Post instillation of Restasis^®^ at 1 h (**b″**); at 3 h (**c″**); at 6 h (**d″**) and at 24 h (**e″**). All other images did not show any redness or abnormal discharge (i.e., indicating the normal features) were represented by green arrows.

No significant signs or symptoms of discomfort were found during the irritation testing in the treated animals with blank micelles (unloaded), CyA-loaded micelles, and Restasis^®^. Figure 10a,a′,a″ represent images of NaCl treated (right) eyes of rabbits of the respective group (green arrows). Figure 10b,b′ represent the mild redness (red arrows) of the conjunctiva and abnormal discharge at 1 h post instillation of blank micelles and CyA-Micelles, respectively. This abnormal discharge might be due to the surfactant property of the di-block copolymer and physiological secretion, which is in agreement with our previous report [21]. No redness or inflammation was observed in the Restasis^®^ treated eyes even at the initial hour (1 h) of the experiment as shown in Figure 10b″ (green arrow). In fact, in the present investigation, the Restasis^®^ treated animals did not show any signs and symptoms of ocular irritation at any time points as represented in Figure 10c″,d″,e″ (green arrow). The redness of conjunctiva was decreased at 3 h post instillation of CyA-Micelles (Figure 10c′) (black arrow) and at 6 h it disappeared (Figure 10d′) (green arrow) and the eyes were recovered their normal conditions at 24 h (Figure 10e″) (green arrow). Redness of conjunctiva was also decreased at 3 h in the blank micelles treated eye (Figure 10c) (black arrow), it disappeared at 6 h (Figure 10d) (green arrow) and the eyes regained their normal condition at 24 h (Figure 10e) (green arrow), that might be due to the natural defensive mechanism of the animal and lower irritation potential of the unloaded Myrj S100-*b*-PCL_88_ polymeric micelles.

As a result of blank micelles instillation, a slight irritation was found in the treated eye of one rabbit with some watery discharge (slightly different from normal but not mucoidal) and it was given score 1. No corneal opacity or lesions in the eye structures was noted in the treated animals. For cornea, iris, and conjunctiva grade-0 was given for all the tested products. Based on a reported classification system for eye irritation scoring [36], the calculated maximum mean total score (MMTS) during the visual examination after 24 h of first dosing for the blank micelles was 19.33 (which is greater than 15.1 but less than 25). The MMTS for CyA-loaded micelles and Restasis^®^ was 9.00 (which is greater than 2.6 but less than 15) (Table 5). Thus, the blank polymeric micelles were considered “mildly irritating” while the CyA-loaded polymeric micelles and the marketed Restasis^®^ were “minimally irritating” to the rabbit eyes in the present investigation.

All the involved animals were active and healthy without any abnormal signs and symptoms of overall toxicity throughout the experiment, except for the observed signs as scored and mentioned in Table 4. No severe eye irritation was found that may arise due to any corneal abrasion related to the treatment or any obstruction in the lacrimal drainage by the CyA-micelles in comparison to Restasis^®^. This indicated that the developed Myrj S100-*b*-PCL_88_ micellar formulation was non-irritant to rabbit eyes. Additionally, during the visual examination after 24 h of the treated eyes, no remaining formulations were observed in the eyes, suggesting a complete degradation or disposition of the applied formulations at 24 h. The overall obtained “minimally irritating” CyA-Micelles in this investigation were in agreement and substantiate with the previous reports, where the block copolymers such (PEO-*b*-PCL) [21] and methoxy poly(ethylene glycol)-hexylsubstituted poly(lactide) (MPEG-hexPLA) [37] micelle carriers were used for topical ocular delivery of CyA. Similarly, polyhydroxyethylaspartamide-polyethylene glycol with hexadecylamine [PHEA-PEG-C_16_] copolymer-based micelles were used for topical ocular delivery of dexamethasone and no irritation was reported [38]. All together, we can conclude that CyA-Micelles were well tolerated by rabbit eyes.

## 4. Conclusions

Myrj S40-*b*-PCL and Myrj S100-*b*-PCL copolymers were successfully synthesized, but only Myrj S100-*b*-PCL formed micelles with optimum size, PDI, and no precipitation. The mean diameters of the prepared self-assembled structures were in the nano-range (≤200 nm). Moreover, Myrj S100-*b*-PCL micelles significantly increased the aqueous solubility of CyA from ca. 23 µg/mL to over 540 µg/mL. The developed micelles showed a transcorneal permeation comparable to Restasis^®^, the leading commercial CyA ocular formulation. The in vivo ocular irritation study demonstrated that CyA-loaded Myrj S100-b-PCL_88_ micelles were well tolerated in the rabbit eye. Our results point to a great potential for Myrj-*b*-PCL micelles to serve as an efficient solubilizing and delivery system for CyA and potentially other hydrophobic drugs.

## Figures and Tables

**Table 1:** Characteristics of the synthesized Myrj-*b*-PCL copolymers.

Block Copolymer ^a^	Theoretical MW (g/mol)	Mn ^b^ (g/mol)	Mn ^c^ (g/mol)	Mw ^d^ (g/mol)	Dispersity ^e^ (Ð)
Myrj S40-*b*-PCL_18_	4100	3500	3007	4037	1.34
Myrj S40-*b*-PCL_35_	6040	5700	4116	5745	1.40
Myrj S100-*b*-PCL_44_	9700	9400	6192	8628	1.39
Myrj S100-*b*-PCL_88_	14,650	14,600	10,150	15,724	1.55
Myrj S100-*b*-PCL_131_	19,700	19,600	12,019	28,880	1.67

^a^ The number shown as a subscript indicates the polymerization degree of each block determined by ^1^H NMR. ^b^ Number-average molecular weight measured by ^1^H NMR. ^c^ Number-average molecular weight measured by GPC. ^d^ Weight-average molecular weight measured by GPC. ^e^ Dispersity (Mw/Mn) determined by GPC.

**Table 2:** Physical characterization of micelles before and after drug loading. Results are expressed as mean with ± SD, *n* = 3.

Polymeric Micelles	Particle Size (nm)	Polydispersity Index	ζ-Potential (mV)	EE (%)	DL (%)
**Micelles before CyA loading**					
Myrj S100-*b*-PCL_44_	49.8 ± 14.9	0.38 ± 0.14	−7.0 ± 1.8	–	–
Myrj S100-*b*-PCL_88_	70.3 ± 2.4	0.20 ± 0.04	−4.7 ± 0.3	–	–
Myrj S100-*b*-PCL_131_	72.6 ± 1.3	0.21 ± 0.02	−9.4 ± 0.7	–	–
**Micelles after CyA loading**					
Myrj S100-*b*-PCL_44_	41.1 ± 5.6	0.44 ± 0.06	−4.9 ± 3.2	15.23 ± 0.38	1.38 ± 0.03
Myrj S100-*b*-PCL_88_	81.9 ± 2.9	0.18 ± 0.01	−6.8 ± 2.1	54.15 ± 3.03	5.62 ± 0.27
Myrj S100-*b*-PCL_131_	63.5 ± 13.1	0.28 ± 0.05	−1.6 ± 1.7	23.04 ± 1.35	2.25 ± 0.13

**Table 3:** Transcorneal permeation parameters for CyA from Myrj S100-*b*-PCL_88_ micelles and Restasis^®^ (Mean ± SD, *n* = 3).

Parameters	CyA-Micelles	Restasis^®^
Cumulative amount of CyA permeated (µg cm^−2^) at 4th h	51.14 ± 5.23	59.49 ± 8.67
Steady-state flux, *J* (µg cm^−2^ h^−1^)	18.89 ± 2.99	17.14 ± 2.79
Permeability coefficient, *P_app_* (cm h^−1^)	(3.78 ± 0.59) × 10^−2^	(3.43 ± 0.56) × 10^−2^

**Table 4:** Weighted scores for eye irritation test by Restasis^®^, blank micelles, and CyA-Micelles.

Lesions in the Treated Eyes	Individual Scores for Eye Irritation								
	Restasis^®^			Blank Micelles			CyA-Micelles		
	Animal #			Animal #			Animal #		
	**1st**	**2nd**	**3rd**	**1st**	**2nd**	**3rd**	**1st**	**2nd**	3rd
**Cornea**									
**I. Opacity (Degree of density)**	0	1	0	0	1	1	0	1	0
**II. Area of cornea**	4	4	4	4	4	4	4	4	4
**Total scores = (I × II × 5) =**	**0**	**20**	**0**	**0**	**20**	**20**	**0**	**20**	**0**
**Iris**									
**I. Lesion values**	0	1	0	0	1	1	0	1	0
**Total scores = (I × 5) =**	**0**	**5**	**0**	**0**	**5**	**5**	**0**	**5**	**0**
**Conjunctiva**									
**I. Redness**	0	0	1	1	1	1	0	1	0
**II. Chemosis**	0	0	0	0	0	0	0	0	0
**III. Mucoidal discharge**	0	0	0	0	1	0	0	0	0
**Total scores = (I + II + III) × 2 =**	**0**	**0**	**2**	**2**	**4**	**2**	**0**	**2**	**0**

**Table 5:** Maximum Mean Total Score (MMTS) calculations for the tested formulations as per the obtained scores in Table S2.

**Restasis^®^**					
**Animal #** **→**	**1st**	**2nd**	**3rd**	**SUM**	**Average (SUM/3)**
**Cornea**	0	20	0	20	6.67
**Iris**	0	5	0	5	1.67
**Conjunctiva**	0	0	2	2	0.66
**SUM total =**	**0**	**25**	**2**	**27**	**9.00**
**Blank micelles**					
**Animal #** **→**	**1st**	**2nd**	**3rd**	**SUM**	**Average (SUM/3)**
**Cornea**	0	20	20	40	13.33
**Iris**	0	5	5	10	3.33
**Conjunctiva**	2	4	2	8	2.67
**SUM total =**	**2**	**29**	**27**	**58**	**19.33**
**CyA-Micelles**					
**Animal #** **→**	**1st**	**2nd**	**3rd**	**SUM**	**Average (SUM/3)**
**Cornea**	0	20	0	20	6.67
**Iris**	0	5	0	5	1.67
**Conjunctiva**	0	2	0	2	0.66
**Total =**	**0**	**27**	**0**	**27**	**9.00**

## Data Availability

Data are available upon request from the corresponding author.

## Supplementary Materials

The following supporting information can be downloaded at: https://www.mdpi.com/article/10.3390/polym14091635/s1, Figure S1. Representative ^1^H NMR spectra of Myrj S40 (A) and Myrj S40-*b*-PCL_35_ (B); Figure S2. GPC chromatograms of unmodified Myrj S40 and the synthesized Myrj S40-*b*-PCL copolymers; Figure S3. FTIR spectra of Myrj S40 (A), Myrj S40-*b*-PCL_18_ (B), and Myrj S40-*b*-PCL_35_ (C); Figure S4. XRD diffractograms of Myrj S40 (A), Myrj S40-*b*-PCL_18_ (B), and Myrj S40-*b*-PCL_35_ (C); Figure S5. DSC thermograms of Myrj S40, Myrj S40-*b*-PCL_18_, and Myrj S40-*b*-PCL_35;_ Figure S6. Representative size distribution profiles of (A) unloaded Myrj S100-*b*-PCL_44_ micelles and (B) CyA-loaded Myrj S100-*b*-PCL_44_ nanocarriers obtained by dynamic light scattering (Zetasizer Nano ZS, Malvern Instrument Ltd., Malvern, UK) showing bimodal size population; Figure S7. Representative size distribution profiles of (A) unloaded Myrj S100-*b*-PCL_88_ micelles and (B) CyA-loaded Myrj S100-*b*-PCL_88_ nanocarriers obtained by dynamic light scattering (Zetasizer Nano ZS, Malvern Instrument Ltd., Malvern, UK) showing bimodal size population; Table S1. Grading system for ocular lesions in irritation tests; Table S2. Classification of eye irritation scoring system.

## References

[1] Rowe R.C., Sheskey P.J., Owen S.C., Association A.P. (2006). Handbook of Pharmaceutical Excipients.

[2] Liu C., Wu J., Shi B., Zhang Y., Gao T., Pei Y. (2006). Enhancing the Bioavailability of Cyclosporine A Using Solid Dispersion Containing Polyoxyethylene (40) Stearate. Drug Dev. Ind. Pharm..

[3] Lo Y.L. (2003). Relationships between the hydrophilic-lipophilic balance values of pharmaceutical excipients and their multidrug resistance modulating effect in Caco-2 cells and rat intestines. J. Control. Release.

[4] Wang S.-W., Monagle J., McNulty C., Putnam D., Chen H. (2004). Determination of P-glycoprotein inhibition by excipients and their combinations using an integrated high-throughput process. J. Pharm. Sci..

[5] Dash T.K., Konkimalla V.B. (2012). Poly-epsilon-caprolactone based formulations for drug delivery and tissue engineering: A review. J Control. Release.

[6] Yusuf O., Ali R., Alomrani A.H., Alshamsan A., Alshememry A.K., Almalik A.M., Lavasanifar A., Binkhathlan Z. (2021). Design and Development of D–α–Tocopheryl Polyethylene Glycol Succinate–block–Poly(ε-Caprolactone) (TPGS-b-PCL) Nanocarriers for Solubilization and Controlled Release of Paclitaxel. Molecules.

[7] Sinha V.R., Bansal K., Kaushik R., Kumria R., Trehan A. (2004). Poly-epsilon-caprolactone microspheres and nanospheres: An overview. Int. J. Pharm..

[8] Woodruff M.A., Hutmacher D.W. (2010). The return of a forgotten polymer—Polycaprolactone in the 21st century. Prog. Polym. Sci..

[9] Aliabadi H.M., Brocks D.R., Lavasanifar A. (2005). Polymeric micelles for the solubilization and delivery of cyclosporine A: Pharmacokinetics and biodistribution. Biomaterials.

[10] Binkhathlan Z., Hamdy D.A., Brocks D.R., Lavasanifar A. (2010). Development of a polymeric micellar formulation for valspodar and assessment of its pharmacokinetics in rat. Eur. J. Pharm. Biopharm..

[11] Shuai X., Merdan T., Schaper A.K., Xi F., Kissel T. (2004). Core-cross-linked polymeric micelles as paclitaxel carriers. Bioconjugate Chem..

[12] Amber T., Tabassum S. (2020). Cyclosporin in dermatology: A practical compendium. Dermatol. Ther..

[13] Airody A., Heath G., Lightman S., Gale R. (2016). Non-Infectious Uveitis: Optimising the Therapeutic Response. Drugs.

[14] Schultz C. (2014). Safety and Efficacy of Cyclosporine in the Treatment of Chronic Dry Eye. Ophthalmol. Eye Dis..

[15] Peng C.C., Bengani L.C., Jung H.J., Leclerc J., Gupta C., Chauhan A. (2011). Emulsions and microemulsions for ocular drug delivery. J. Drug Deliv. Sci. Technol..

[16] Binkhathlan Z., Alomrani A.H., Alshamsan A., Aljuffali I.I., Ali R. (2017). Poly e-Caprolactone-Ethoxylated Fatty Acid Copolymers. U.S. Patent.

[17] Topel Ö., Çakır B.A., Budama L., Hoda N. (2013). Determination of critical micelle concentration of polybutadiene-block-poly(ethyleneoxide) diblock copolymer by fluorescence spectroscopy and dynamic light scattering. J. Mol. Liq..

[18] Soleymani Abyaneh H., Vakili M.R., Zhang F., Choi P., Lavasanifar A. (2015). Rational design of block copolymer micelles to control burst drug release at a nanoscale dimension. Acta Biomater..

[19] Binkhathlan Z., Ali R., Qamar W., Lavasanifar A. (2018). Pharmacokinetics of Orally Administered Poly(Ethylene Oxide)-block-Poly(ε-Caprolactone) Micelles of Cyclosporine A in Rats: Comparison with Neoral^®^. J. Pharm. Pharm. Sci..

[20] Al-Jenoobi F.I., Alam M.A., Alkharfy K.M., Al-Suwayeh S.A., Korashy H.M., Al-Mohizea A.M., Iqbal M., Ahad A., Raish M. (2014). Pharmacokinetic interaction studies of fenugreek with CYP3A substrates cyclosporine and carbamazepine. Eur. J. Drug Metab. Pharmacokinet..

[21] Alshamsan A., Abul Kalam M., Vakili M.R., Binkhathlan Z., Raish M., Ali R., Alturki T.A., Safaei Nikouei N., Lavasanifar A. (2019). Treatment of endotoxin-induced uveitis by topical application of cyclosporine a-loaded PolyGel in rabbit eyes. Int. J. Pharm..

[22] Kalam M.A., Alshamsan A. (2017). Poly (d, l-lactide-co-glycolide) nanoparticles for sustained release of tacrolimus in rabbit eyes. Biomed. Pharmacother..

[23] Draize J.H., Woodard G., Calvery H.O. (1944). Methods for the study of irritation and toxicity of substances applied topically to the skin and mucous membranes. J. Pharmacol. Exp. Ther..

[24] Lee M., Hwang J.H., Lim K.M. (2017). Alternatives to In Vivo Draize Rabbit Eye and Skin Irritation Tests with a Focus on 3D Reconstructed Human Cornea-Like Epithelium and Epidermis Models. Toxicol. Res..

[25] Gyun Shin I.L., Yeon Kim S., Moo Lee Y., Soo Cho C., Yong Kiel S. (1998). Methoxy poly(ethylene glycol)/ϵ-caprolactone amphiphilic block copolymeric micelle containing indomethacin.: I. Preparation and characterization. J. Control. Release.

[26] Sun J., He C., Zhuang X., Jing X., Chen X. (2011). The crystallization behavior of poly(ethylene glycol)-poly(ε-caprolactone) diblock copolymers with asymmetric block compositions. J. Polym. Res..

[27] Sharma R., Murali R., Murthy C.N. (2012). Clouding and Aggregation Behavior of PPO-PEO-PPO Triblock Copolymer (Pluronic^®^25R4) in Surfactant Additives Environment. Tenside Surfactants Deterg..

[28] Mahmud A., Xiong X.-B., Lavasanifar A. (2006). Novel Self-Associating Poly(ethylene oxide)-*block*-poly(ε-caprolactone) Block Copolymers with Functional Side Groups on the Polyester Block for Drug Delivery. Macromolecules.

[29] Atanase L.I., Winninger J., Delaite C., Riess G. (2014). Micellization and demicellization of amphiphilic poly(vinyl acetate)-graft-poly(N-vinyl-pyrrolidone) graft copolymers in the presence of sodium dodecyl sulfate. Colloids Surf. A Physicochem. Eng. Asp..

[30] Fairley N., Hoang B., Allen C. (2008). Morphological Control of Poly(ethylene glycol)-*block*-poly(ε-caprolactone) Copolymer Aggregates in Aqueous Solution. Biomacromolecules.

[31] Qi W., Ghoroghchian P.P., Li G., Hammer D.A., Therien M.J. (2013). Aqueous self-assembly of poly(ethylene oxide)-*block*-poly(ε-caprolactone) (PEO-*b*-PCL) copolymers: Disparate diblock copolymer compositions give rise to nano- and meso-scale bilayered vesicles. Nanoscale.

[32] Danaei M., Dehghankhold M., Ataei S., Hasanzadeh Davarani F., Javanmard R., Dokhani A., Khorasani S., Mozafari M.R. (2018). Impact of Particle Size and Polydispersity Index on the Clinical Applications of Lipidic Nanocarrier Systems. Pharmaceutics.

[33] Lallemand F., Schmitt M., Bourges J.L., Gurny R., Benita S., Garrigue J.S. (2017). Cyclosporine A delivery to the eye: A comprehensive review of academic and industrial efforts. Eur. J. Pharm. Biopharm..

[34] Kennah H.E., Hignet S., Laux P.E., Dorko J.D., Barrow C.S. (1989). An objective procedure for quantitating eye irritation based upon changes of corneal thickness. Fundam. Appl. Toxicol..

[35] Falahee K.J., Rose C.S., Olin S.S. (1981). Eye Irritation Testing: An Assessment of Methods and Guidelines for Testing Materials for Eye Irritancy.

[36] Kay J.H., Calandra J.C. (1962). Interpretation of eye irritation tests. J. Soc. Cosmet. Chem..

[37] Di Tommaso C., Torriglia A., Furrer P., Behar-Cohen F., Gurny R., Moller M. (2011). Ocular biocompatibility of novel Cyclosporin A formulations based on methoxy poly(ethylene glycol)-hexylsubstituted poly(lactide) micelle carriers. Int. J. Pharm..

[38] Civiale C., Licciardi M., Cavallaro G., Giammona G., Mazzone M.G. (2009). Polyhydroxyethylaspartamide-based micelles for ocular drug delivery. Int. J. Pharm..

