# Supplementary material for: Development and Characterization of PEGylated Fatty Acid-Block-Poly(ε-caprolactone) Novel Block Copolymers and Their Self-Assembled Nanostructures for Ocular Delivery of Cyclosporine A
Source: Polymers (Basel). 2022 Apr 19;14(9):1635. doi: 10.3390/polym14091635 (PMC9101097; doi:10.3390/polym14091635)
Supplement: Supplementary file 1 [file polymers-14-01635-s001.zip › Supplementary Materials.pdf]

Supplementary Materials:

Development and Characterization of PEGylated Fatty Acid-*block*-Poly(ε-Caprolactone) Novel Block Copolymers and Their Self-Assembled Nanostructures for Ocular Delivery of Cyclosporine A

Ziyad Binkhathlan*, Abdullah H. Alomrani, Olsi Hoxha, Raisuddin Ali, Mohd Abul Kalam, and Aws Alshamsan

Supplementary Figures

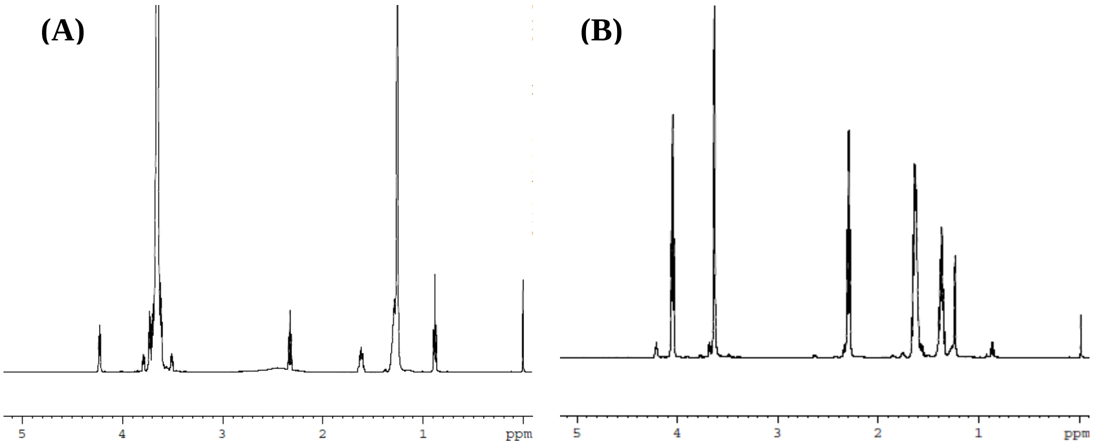

Figure S1. Representative ^{1}H NMR spectra of Myrj S40 (A) and Myrj S40-*b*-PCL_{35} (B).

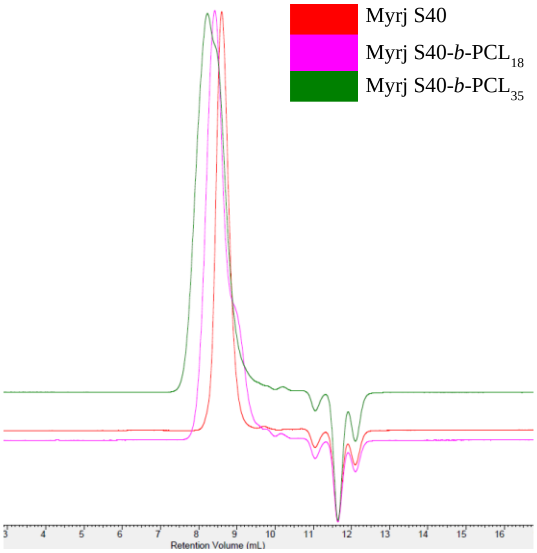

Figure S2. GPC chromatograms of unmodified Myrj S40 and the synthesized Myrj S40-*b*-PCL copolymers.

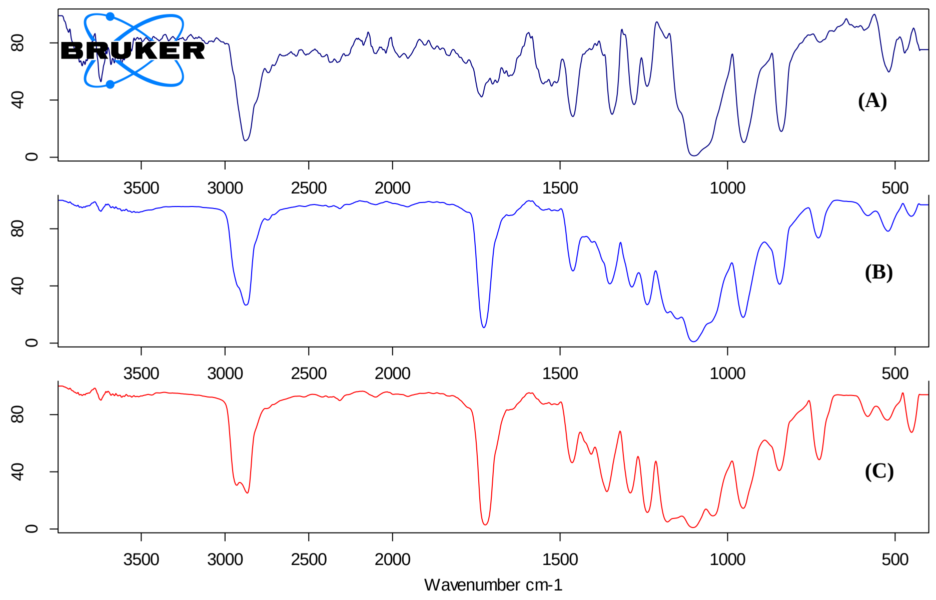

Figure S3. FTIR spectra of Myrj S40 (A), Myrj S40-*b*-PCL$_{18}$ (B), and Myrj S40-*b*-PCL$_{35}$ (C).

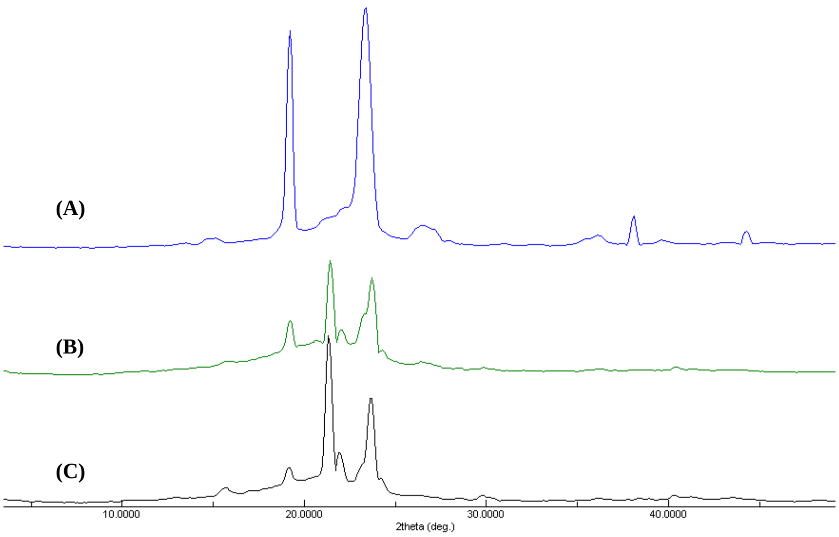

Figure S4. XRD diffractograms of Myrj S40 (A), Myrj S40-*b*-PCL$_{18}$ (B), and Myrj S40-*b*-PCL$_{35}$ (C).

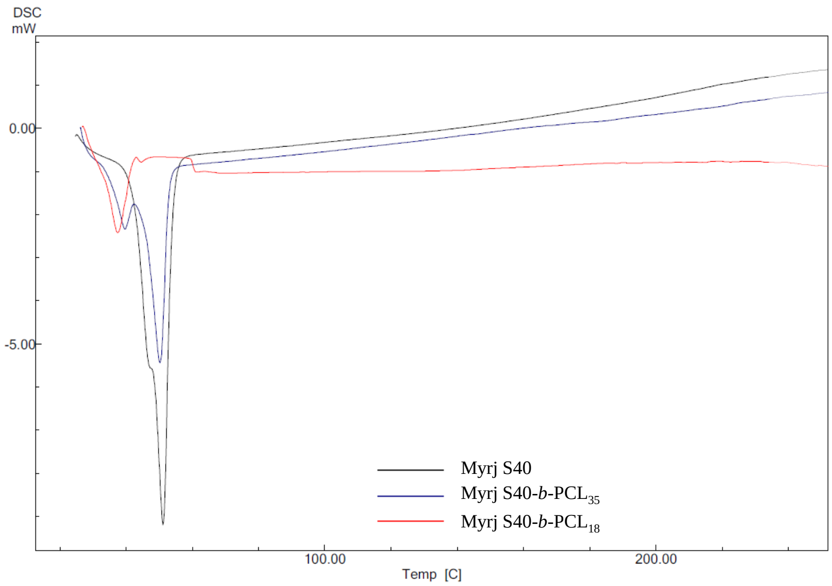

Figure S5. DSC thermograms of Myrj S40, Myrj S40-*b*-PCL_{18}, and Myrj S40-*b*-PCL_{35}.

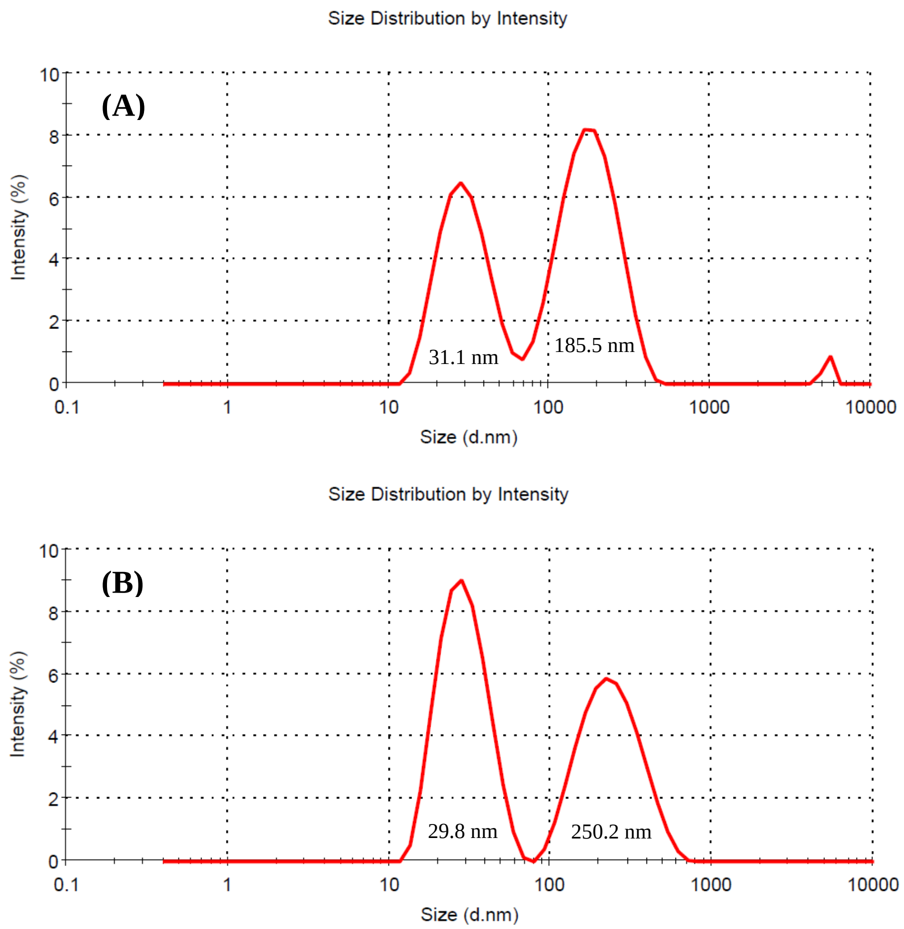

Figure S6. Representative size distribution profiles of (A) unloaded Myrj S100-*b*-PCL_{44} micelles and (B) CyA-loaded Myrj S100-*b*-PCL_{44} nanocarriers obtained by dynamic light scattering (Zetasizer Nano ZS, Malvern Instrument Ltd., UK) showing bimodal size population.

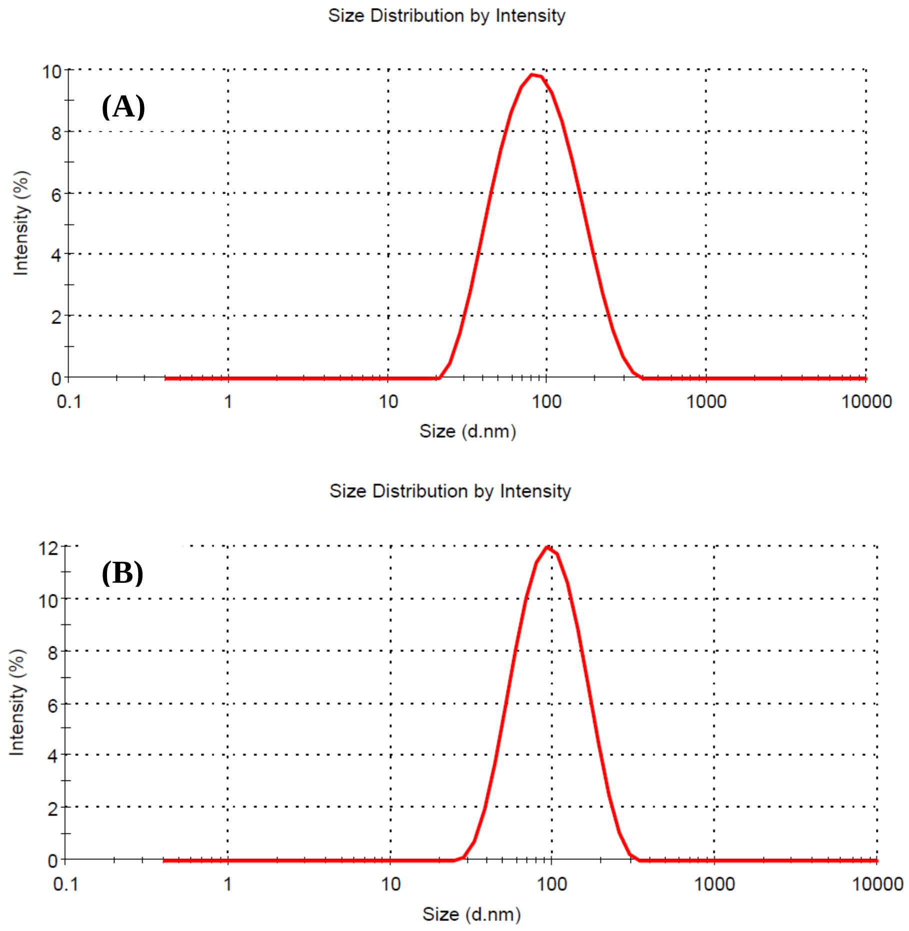

Figure S7. Representative size distribution profiles of (A) unloaded Myrj S100-*b*-PCL_{88} micelles and (B) CyA-loaded Myrj S100-*b*-PCL_{88} nanocarriers obtained by dynamic light scattering (Zetasizer Nano ZS, Malvern Instrument Ltd., UK) showing bimodal size population.

Supplementary Tables

Table S1. Grading system for ocular lesions in irritation tests

Cornea		
**Lesion**		**Score**
**I. Opacity-Degree of density (area which is most dense is taken for reading)**		
No ulceration or opacity		**0**
Scattered or diffuse area – details of iris clearly visible		**1**
Easily discernible translucent areas, details of iris slightly obscured		**2**
Opalescent areas, no details of iris visible, size of pupil barely discernible		**3**
Opaque, iris invisible		**4**
**II. Area of cornea involved**		
One quarter (or less) but not zero		**1**
Greater than one quarter but less than one half		**2**
Greater than one half but less than three quarters		**3**
Greater than three quarters up to whole area		**4**
**Score equals (I x II x 5):**	**Total maximum =**	**80**
**Iris**		
**Lesion**		**Score**
**I. Values**		
Normal		**0**
Folds above normal, congestion, swelling, circumcorneal injection (any one or all of these or combination of any thereof), iris still reacting to light (sluggish reaction is positive)		**1**
No reaction to light, hemorrhage; gross destruction (anyone/ all of these)		**2**
**Score equals (I x 5):**	**Total maximum =**	**10**
**Conjunctiva**		
**Lesion**		**Score**
**I. Redness (refers to palpebral conjunctiva only)**		
Vessels normal		**0**
Vessels definitely injected above normal		**1**
More diffuse, deeper crimson red, individual vessels not easily discernible		**2**
Diffuse beefy red		**3**
**II. Chemosis**		
No swelling		**0**
Any swelling above normal (includes nictitating membrane)		**1**
Obvious swelling with partial eversion of the lids		**2**
Swelling with lids about half closed		**3**
Swelling with lids about half closed to completely closed		**4**
**III. Discharge**		
No discharge		**0**
Any amount different from normal (does not include small amount observed in inner canthus of normal animals)		**1**
Discharge with moistening of the lids and hairs just adjacent to the lids		**2**
Discharge with moistening of the lids and considerable area around the eye		**3**
**Score equals (I + II + III) x 2:**	**Total maximum =**	**20**

Table S2. Classification of eye irritation scoring system

**MMTS***	**Irritation classification**
0.0-0.5	None
0.6-2.5	Practically none
2.6-15.0	Minimally
15.1-25.0	Mildly
25.1-50.0	Moderately
50.1-80.0	Severely
80.1-100.0	Extremely
100.1-110.0	Maximally

* MMTS: Maximum Mean Total Score
